# Liver Fibrosis and Purinergic Signaling: Autocrine–Paracrine Role of ATP in Liver Damage

**DOI:** 10.3390/ijms27136030

**Published:** 2026-07-05

**Authors:** Blanca Verónica Ramos-Rosillo, Esperanza Mata-Martínez, Mauricio Díaz-Muñoz, Francisco G. Vázquez-Cuevas

**Affiliations:** 1 Departamento de Neurobiología Celular y Molecular, Instituto de Neurobiología, Universidad Nacional Autónoma de México, Juriquilla, Querétaro CP 76230, Mexico; veroramos9615@gmail.com; 2 Departamento de Biología Celular, Instituto de Fisiología Celular, Universidad Nacional Autónoma de México, Ciudad de México CP 04510, Mexico; espemmtz@gmail.com

**Keywords:** extracellular matrix, liver fibrosis, Kupffer cells, hepatic stellate cells, hepatocytes, purinome, purinergic receptors, P2X7, P2Y2

## Abstract

Fibrosis is a common extracellular matrix pathology characterized by increased scarring, representing a critical checkpoint toward cirrhosis and hepatocellular carcinoma. Its onset involves coordinated interplay among hepatocytes, Kupffer, and hepatic stellate cells (HSCs). Extracellular ATP and its derivates act as crucial damage-associated molecular patterns when released by injured liver cells, binding to specific purinergic receptors (P2X, P2Y, and P1) to establish an autocrine–paracrine signaling loop. The hepatic fibrotic response underlies the activation of ATP receptors that generate second messengers and cationic conductance. In parallel, extracellular nucleotidases hydrolyze ATP towards less phosphorylated intermediates and adenosine. This review focuses on the role of P2X and P2Y receptors in liver injury. The P2X7 receptor regulates the NLRP3 inflammasome in Kupffer cells and HSCs, while the P2X4 receptor is upregulated in myofibroblasts, modulating migration and matrix synthesis. Among P2Y receptors, P2Y2 drives inflammation and steatosis but promotes HIF-1α-mediated DNA repair. The P2Y6 receptor promotes alcohol-induced injury but restrains metabolic-dysfunction-associated steatohepatitis. P2Y2 and P2Y4 receptors maintain biliary homeostasis in cholangiocytes, whereas the P2Y1 receptor preserves HSC quiescence by blocking YAP translocation. Finally, UDP-glucose–P2Y14 induces HSC activation. Targeting these specific purinergic receptors or ecto-nucleotidases represents a promising pharmacological frontier against hepatic fibrosis.

## 1. Introduction

### 1.1. Chronic Liver Disease and Metabolic-Dysfunction-Associated Steatotic Liver Disease

In 2023, the term non-alcoholic fatty liver disease (NAFLD) was replaced by metabolic-dysfunction-associated steatotic liver disease (MASLD). Therefore, the disease is no longer diagnosed by exclusion (ruling out alcohol) but by affirmative diagnosis; that is, based on metabolic parameters such as overweight, dyslipidemias, cardiovascular anomalies, and diabetes [1]. The pathognomonic characteristic of MASLD is the accumulation of triacylglycerides within hepatocytes, a condition known as metabolic-dysfunction-associated steatohepatitis (MASH), previously termed non-alcoholic steatohepatitis (NASH). The severity of liver conditions depends on the extent of scarring (i.e., fibrosis) and the degree of the inflammatory response, both of which alter hepatic function. Fibrosis occurs in four stages, from stage 0 (mild fatty liver) to stage 4 in which fibrosis is serious but still reversible. The onset of fibrosis and its physiopathological outcomes is a very complex and context-dependent process. The principal factor that promotes the progression from cellular damage to fibrosis, cirrhosis, and cancer is the persistence of harmful stimuli. In the liver, hepatocytes are the cells most commonly affected by pathogens, toxics, autoimmune diseases, and nutritional influences. Damaged hepatocytes communicate the stressful condition to the rest of the hepatic cells by several strategies, but one of the most relevant is the release of DAMPs to the extracellular space. Among DAMPs, ATP and UDP/glucose orchestrate the coordinated response of Kupffer cells (KCs), hepatic stellate cells (HSCs), biliary epithelial cells, and liver sinusoidal endothelial cells (LSECs). ATP and other nucleotides recognize a set of receptors (ion channels, P2X, and GPCRs, P2Y) in each cellular type activating signaling pathways that implement a global inflammatory response. A major step in this process is transdifferentiation that favors the phenotypic change from quiescent HSCs (qHSCs) to active fibrogenic myofibroblasts (MFs), which are the principal fibrogenic cells in the liver. In the presence of TGF-β, MFs enhance the formation of the extracellular matrix by promoting the synthesis of collagen I and III, fibronectin, and proteoglycans, as well as the reduction in metalloproteinase activities (MMPs) by the formation of their inhibitors (TIMPs). The fibrogenetic event is further supported by inflammatory intermediates secreted by Kupffer cells and infiltrated monocytes. Eventually, the upgraded extracellular matrix favors the formation of fibrous bands of scarring material that promote hepatic hypoxic conditions, increasing the expression of HIF-1α and the structural and metabolic alteration of liver parenchyma. In parallel, sinusoids lose their fenestrations, reducing the molecular exchange between the liver and circulating factors, the portal hypertension increases, and the onset and development of regenerative nodules are favored. As a result of this pathological process, the liver presents fibrotic septa, regenerative nodules with parenchymal hepatocytes deeply affected by stiffness conditions and severe oxidative stress. Now, the liver has become cirrhotic, and the organism is prone to present exacerbated portal hypertension, liver failure, and the risk of developing hepatocarcinoma [2,3,4]. Episodes of fibrosis involve specific characteristics of each affected system, and they depend on particular causal factors and triggers. Indeed, fibrosis is an important link related with a variety of relevant translational hepatic pathologies; however, the present review is focused mainly on the early events associated with hepatic fibrosis.

### 1.2. Fibrosis

Fibrosis is defined as the excessive accumulation of protein elements in the extracellular matrix (ECM). The ECM components play a physiological role in the coordinated communication among cellular types in a given tissue or organ. However, when connective proteins, such as collagens, fibronectin, elastin, laminin, and proteoglycans, are pathogenically accumulated they indicate a fibrotic process, characterized as an organic affliction that compromises cellular function [5]. The occurrence of liver fibrosis is linked to a condition of persistent cellular stress, usually related to illness (virus), intake of organic chemicals (such as alcohol), or nutritional imbalance (associated with MASLD). One of the pivotal processes leading to fibrosis is oxidative stress; in this event, NADPH oxidases (NOXs) play a relevant role (reviewed in [6]).

Fibrotic response is context-dependent, but it usually promotes changes in substrate stiffness and tissue architecture, impairment of sinusoidal exchange, incremental increase in vascular resistance, and generation of regenerative nodules. The pathological development of hepatic fibrosis requires coincidental activity among several cell types. HSCs play a central role in this process [7]. HSCs, located in the Space of Disse, are specialized pericytes rich in vitamin A droplets. Under basal conditions, qHSCs regulate sinusoidal blood flow and produce diverse molecular messengers. However, when hepatic tissue is injured, they react by sensing the release of chemical intermediates (mislocalized metabolites; see next section) from hepatocytes and KCs; transforming growth factor-β [TGF-β], platelet-derived growth factor [PDGF], and pro-inflammatory cytokines), leading to the phenotypic transformation of HSCs into MFs. MFs are responsible for the synthesis and accumulation of new ECM material, namely fibronectin, proteoglycans, and collagen 1 and 3, in the onset of the fibrotic liver [8]. It is appropriate to mention that fibrotic response may eventually compromise the integrity of all liver cell populations, resulting in a potential ATP release from each cellular type. ATP is not only released by necrotic cells; other forms by which ATP reaches the extracellular medium from damaged cells are pannexin and connexin channels, ABC transporters, and the purinergic receptor P2X7.

### 1.3. Reversibility of Fibrosis

Reversibility of liver fibrosis upon the removal of the causative agent has been documented in patients [9,10,11,12,13] and experimental fibrosis models [14,15,16,17]. This reversibility is characterized by a decrease in pro-inflammatory factors, including interleukin (IL)-6, IL-1β, tumor necrosis factor-alpha (TNF-α), and TGF-β. In the absence of TGF-β signaling, MFs rapidly decline and ECM overproduction is abrogated. Furthermore, the expression of matrix metalloproteinases (MMPs) that degrade collagen fibers is upregulated, surpassing the concentrations of their inhibitors (tissue inhibitors of metalloproteinase [TIMP] proteins) [18]. During the resolution of liver fibrosis, MFs are susceptible to apoptosis and can undergo senescence and death-receptor-mediated cell death caused by deprivation of fibrogenic cytokines [15,19,20].

Upon the withdrawal of fibrogenic signals, MFs increase the expression of the Fas receptor or TNF-α receptor 1 and their ligands, and undergoes a process of caspase-8/caspase-3-dependent apoptosis. Alternatively, shifting the balance toward pro-apoptotic proteins (e.g., p53, Bax) activates the intrinsic pathway, resulting in caspase-9-mediated programmed cell death [14]. Furthermore, natural killer cells and liver-specific ϒ**δ** T cells, activated by interferon-ϒ, induce a rapid decrease in MFs [20,21,22].

On the other hand, it has been demonstrated that approximately half of hepatic MFs escape apoptosis after cessation of liver injury. These MFs return to the Space of Disse and revert to an inactivated phenotype (inactivated hepatic stellate cells, iHSCs) [17,23]. The expression of fibrogenic genes (*COL1A1* and *α-SMA*) in iHSCs is reduced, whereas the expression of some quiescence-associated genes, such as *PPARγ*, is increased to similar amounts as those detected in qHSCs. However, some genes associated with qHSCs, including *GFAP*, *ADIPOR1*, *ADPF*, and *DBP*, are not re-expressed in iHSCs [17,24]. Moreover, compared to original qHSCs, iHSCs are more responsive to fibrogenic stimuli and can contribute to recurring liver fibrosis more effectively [17,24]. Additionally, the resolution of fibrosis is associated with a dynamic renewal of HSCs that emerge in the recovering liver from an unidentified source, and which possess all the characteristics of qHSCs [17,23].

Another important component of hepatic fibrosis regression is macrophage conversion. Macrophages play dual roles throughout liver fibrosis progression and resolution. Resident KCs are CD68^+^; MFs express *CD5L*, *CD163*, *TIMD4,* and *VSIG4* while other resident non-KCs and MFs are also positive for CD68 but lack the other markers [25]. During the progression of fibrosis, macrophages are recruited to the liver as a consequence of the injury-induced inflammatory response and produce cytokines and chemokines that trigger the transition of qHSCs into pro-fibrotic MFs. CC-motif chemokine ligand 2 (CCL2) is secreted by HSCs and KCs to facilitate the recruitment of immature monocyte-derived Ly6C^hi^ macrophages into the liver [26]. Ly6C is often transient; monocyte-derived KCs express all KC markers except TIM4 [25]. Pro-inflammatory macrophages are positive to CD86 and MHCII, while reparative MFs are positive to CD163 and CD206 [27]. Monocytes recruited during steatosis and fibrosis appear to have a unique identity, and have received distinct names such as lipid-associated macrophages (LAMs), scar-associated macrophages (ASMs), and NASH-associated macrophages (NAMs). All three MFs express a similar expression of markers [28]. A detailed description of MFs in fibrosis has been previously reviewed [28]. Interestingly, deletion of macrophages in the CD11b-DTR transgenic mouse line, which carries a diphtheria toxin-inducible system that depletes macrophages [29], led to reduced scarring and MFs in CCl_4_-induced liver fibrosis, supporting the role of macrophages in promoting this disease [29]. However, during liver fibrosis recovery, macrophages change to a Ly6C^low^ phenotype and stop producing fibrogenic and inflammatory factors; instead, they secrete MMPs, such as MMP9 and MMP12 [30]. MMPs are the primary enzymes capable of ECM degradation [14]. Additionally, MFs are a major source of TIMP production. The disappearance of MFs leads to reduced TIMP levels, increased MMP activity, and ECM degradation [31].

Because reversibility of liver fibrosis depends on the collagenolytic activity of ECM-degrading MMPs, sustained expression of TIMPs inhibits active MMP function. Moreover, the lack of ECM degradation may be caused by tissue transglutaminase, which prevents MF apoptosis and mediates ECM cross-linking. The latter prevents proteolytic cleavage of different collagen types [15,20,32].

### 1.4. Animal Models of Liver Fibrosis

Animal models of liver fibrosis are essential for investigating the pathogenesis of this disease in experimental conditions. There are currently five types of in vivo models: (a) chemical, (b) dietary, (c) surgical, (d) immune, and (e) transgenic animal models [33,34]. The most frequently utilized organisms are mice [35], rats [36], rabbits [37], Ossabaw pigs [37,38], macaques [37,38,39], and zebrafish [40]. Table 1 describes the most common experimental protocols of liver fibrosis, each one offering varying levels of efficacy and contributing differently to the overall understanding of this disease.

### 1.5. Epidemiology

Chronic liver diseases are among the leading causes of death worldwide. According to the 2019 Global Burden of Disease study, mortality related to cirrhosis and other liver diseases increased by 13% between 1990 and 2019 [61]. As the final clinical stage of liver disease, cellular hepatocarcinoma is responsible for 8.3% of global cancer mortality, as reported in the 2020 Global Cancer Statistics update [62]. Viral hepatitis B and C annually cause around 1.3 million deaths [63]. Moreover, approximately 3.3 million people are diagnosed with alcohol-associated liver disease (ALD) per year, accounting for 5.9% of global deaths [64]. The rising fatalities from MASLD are also noteworthy, with an estimated 280,000 deaths in 2019 [65].

Regardless of the cause of liver disease, liver-related mortality increases exponentially with fibrosis [66]. In ALD, severe fibrosis prior to developing cirrhosis has a major impact on 10-year mortality [67]. In MASLD, higher stages of fibrosis are proven to be the strongest predictors of mortality [67,68,69,70].

Otherwise, a recent systematic review and meta-analysis have revealed a considerable rise in the prevalence of advanced liver fibrosis from before 2010 (2.0%) to after 2016 (4.7%). That study also found that advanced fibrosis was significantly more prevalent in men than in women, as well as in older individuals than in younger ones. Furthermore, the prevalence rates varied significantly across continents and countries. Advanced fibrosis had a prevalence of 4.0% in Africa, 2.6% in Asia, 3.4% in Europe, 2.2% in Latin America and the Caribbean, 4.8% in North America, and 10.6% in Oceania [71]. In this context, the study revealed that among the factors potentially associated with the risk of advanced liver fibrosis in the general population, viral hepatitis had the strongest association. Age, diabetes, excessive alcohol intake, hepatic steatosis, obesity, and sex were found to be other likely risk factors for advanced fibrosis [71].

## 2. Purinergic Signaling as a Damage-Associated Molecular Pattern

### 2.1. Damage-Associated Molecular Patterns

The correct activity of biological systems involves functional communication and coordination among their tissular and cellular components. There are several facets to sustaining this homeostatic state, such as endocrine, paracrine, autocrine, and juxtacrine signaling, which allow physiological recognition between ligands and receptors during cellular communication [72]. Another modality in this cellular dialog is the coordinated response elicited in situations of stress, damage, or cellular death, when endogenous molecules lose their compartmentalization and act as damage-associated molecular patterns (DAMPs). DAMPs are released from stressed cells and selectively recognized by pattern recognition receptors (PRRs). A similar response occurs when the molecules acting as PRR ligands are exogenous or come from pathogens such as bacteria, viruses, and other microbes. In this case, the ligands are called PAMPs (pathogen-associated molecular patterns); examples of PAMPs include lipopolysaccharides (LPSs) and peptidoglycans [73].

DAMPs are classified into three principal categories: protein-based, nucleic-acid-based, and mitochondria-derived. The most important PRRs are Toll-like receptors (TLRs), nucleotide-binding oligomerization domain-like receptors (NLRs), C-type lectin receptors (CLRs), and receptors for advanced glycation end-products (RAGEs), whereas the major signaling pathways associated with DAMP responses are NF-κB, MAPK, inflammasomes, and cyclic GMP-AMP synthase-stimulator of interferon genes (cGAS-STING) [74]. Overall, DAMPs and PAMPs promote an alert response that triggers the reversal and repair of cellular damage, and it is usually accompanied by an escalated inflammatory reaction [72].

The variety of DAMPs is extensive. Intracellular molecules that are accepted as DAMPs are: (1) nuclear proteins, like histones and the high mobility group box 1 protein (HMGB1); (2) DNA and RNA fragments, both nuclear and mitochondrial; (3) heat-shock proteins; (4) hyaluronan pieces; and (5) purines, such as uric acid and ATP. The properties and characteristics of ATP acting as a DAMP will be developed in the next section.

### 2.2. ATP

ATP is a multifunctional molecule and the most abundant purine nucleotide in the intracellular environment. ATP supports key cellular processes as a metabolic switch coordinating anabolic and catabolic reactions. It functions as a strategic energy currency in a dynamic equilibrium with less phosphorylated intermediates (ADP, AMP, and adenosine [ADO]) [75]. Within the intracellular milieu, ATP is one of the most concentrated molecules, being present in the 5–10 mM range [76]. It acts as the primary phosphorylating factor during biochemical energy transfer reactions in the cytoplasm and organelles. ATP also serves as an allosteric agent in metabolic pathways related to the synthesis and degradation of energy molecules (mainly sugars and fats) [77]. In contrast, the extracellular presence of ATP is very discreet (30–100 nM), as it is approximately eight orders of magnitude lower than its intracellular concentration. However, most cellular systems can use ATP as a molecular messenger. In those circumstances, ATP is released and reaches temporal concentrations near the low µM range in the extracellular space. ATP acting as a ligand in this context can recognize a variety of receptors, making it a substantial part of purinergic signaling communication [78].

### 2.3. Purinergic Signaling

Purinergic signaling is an interesting cellular communication system that presents two modalities: (1) ATP and other nucleotides recognize G-protein-coupled receptors and ligand-gated ion channels; (2) ADO, which is obtained by ATP degradation, acts as a ligand of a different family of G-protein-coupled receptors [79]. Purinergic receptors activated by ATP are known as P2, whereas receptors activated by ADO are termed as P1. ADO receptor subtypes are A1, A2A, A2B, and A3. Ligands for P2 receptors include ATP, ADP, UTP, UDP, and UDP-glucose, whereas P2 receptors are subdivided into P2X and seven subunits (P2X1–7), forming homo- or hetero-trimeric functional channels whose main conductance mobilizes Na^+^, Ca^2+^ and K^+^, and P2Y (including eight subtypes: P2Y1, 2, 4, 6, 11, 12, 13, and 14). This activity influences intracellular Ca^2+^ and cyclic adenosine monophosphate (cAMP) amounts [80]. Therefore, purine communication involves a complex equilibrium among the expressed ATP and ADO receptors, the activity of different extracellular ectonucleotidases, and the resulting proportion of ATP-ADP and ADO acting as ligands. The principal ectonucleotidases are CD39 and CD73; both can be regulated by chemical messengers such as TGF-β and by hypoxic conditions. The concept that encompasses and controls this dynamic molecular network is known as the purinome [81]. In the context of liver fibrosis, the purinome characteristics define whether the tissue response is inflammatory, fibrogenic, reparative, or immunosuppressive.

The ATP affinity constant (Kd) for P2Y receptors is in the low µM range (0.1–5 µM), whereas the affinity of ATP for P2X receptors is slightly higher (Kd from 5 to 20 µM), with the exception of the P2X7 receptor (see next section), since the ATP binding for this receptor shows a very high Kd of ~500 µM [82]. Interestingly, during severe cellular stress and damage, ATP is released in abundance, causing a significant increase in extracellular ATP concentrations to 10–1000 µM; under these circumstances, ATP acts as a DAMP [74]. Therefore, the P2X7 receptor plays a strategic role in cellular adaptations under stress conditions when ATP is an active DAMP [83].

The crystallographic structure of P2X receptors confirmed that functional receptors are formed by three subunits [84]. All cloned subunits, with the exception of P2X6, may form homotrimers, and all subunits, except P2X7, form heterotrimeric channels [85,86].

## 3. Purinergic Receptors and Liver Fibrosis

Several receptors have been identified as important in the purinergic regulation of hepatic pathologies. In this section, the most representative will be mentioned with respect to their physiopathology and pharmacological actions. However, it should be noted that some receptors are likely harmful and targetable, such as P2X7 receptor inflammasome-driven injury or the P2Y14 receptor in HSC activation, whereas others may have context-dependent or protective effects, such as the P2Y1 receptor delaying HSC activation or the P2Y13 receptor regulating the adipose tissue–liver axis.

### 3.1. P2X7 Receptor

The P2X7 receptor was originally described as a bifunctional protein expressed in immune cells, an ATP-gated channel mediating Ca^2+^/Na^+^ influx and K^+^ efflux, and a cytotoxic receptor characterized by its ability to open the “megapore” (a non-selective and irreversible conductance mediating massive entry of Ca^2+^), leading to the activation of the mitochondria-induced apoptotic pathway [87]. This characteristic motivated its extensive characterization in multiple cellular systems.

Recently, the ability of the ATP/P2X7 receptor to function as a DAMP ligand/receptor pair that modulates the inflammatory process by regulating NLRP3 inflammasome assembly has opened a new avenue in the understanding of the physiological role of the P2X7 receptor, highlighting its relevance in chronic inflammation processes triggered by tissue damage [83]. In this context, we will review the role of the P2X7 receptor in the fibrotic stage of chronic liver disease.

Hepatotoxic damage induced by four weeks of CCl_4_ administration is a well-established model of reversible liver fibrosis [88]. In whole-liver lysates, this treatment increased the expression level of the P2X7 receptor, inflammatory mediators, and the activity of the transcription factor NF-kB [89]. All the effects promoted by CCl_4_ were attenuated by systemic administration of A438079, a potent P2X7 receptor antagonist, suggesting a profibrotic role of this entity in the onset of hepatotoxic liver fibrosis [89].

**Figure 1:**
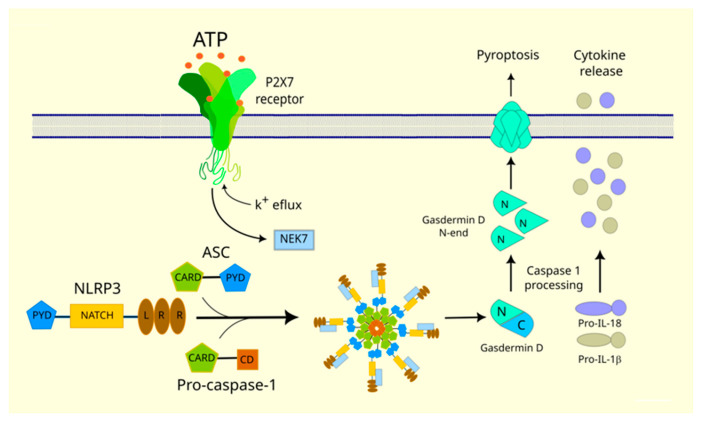
Assembly of NLRP3 inflammasome mediated by ATP/P2X7 receptor. Activation of the P2X7 receptor induces K^+^ efflux, leading to NEK7 kinase activation followed by the assembly of NLRP3 and accessory protein complex. Into the complex, pro-caspase-1 is matured to process pro-IL-18 and pro-IL-1β, and Gasdermin D to split the amino-end (N) from the carboxi-end (C). Apoptosis-associated speck-like protein (ASC) is composed of the CARD and PYD domains and contains a caspase recruitment domain (CARD) and a PYD domain for homotypic interaction with a PYD-containing NLRP3 protein. Procaspase-1 features CARD and caspase (CD) domains; CARD domain mediate homotypic interactions with ASC to recruit procaspase-1 to the inflammasome complex, NLRP3 activation promotes pyroptosis and cytokine release.

The role of sterile inflammation in hepatic disease was studied in a mouse model of alcoholic steatohepatitis (ASH), particularly with ATP and uric acid acting as DAMPs. Null mice for *P2rx7 (P2rx7^−^/^−^),* transgenic mice with increased activity of uric acid metabolism, or mice treated with drugs that reduced the extracellular amounts of ATP and uric acid, showed attenuated liver damage induced by the Lieber–DeCarli alcohol diet in comparison with control mice. All the mentioned experimental models showed reduced inflammasome activity markers, including activated caspase-1 and release of IL-18. Results from in vitro experiments suggested that ATP and uric acid were released by damaged hepatocytes but not by liver mononuclear cells to promote NLRP3 inflammasome assembly [90] (Figure 1).

On the other hand, Blassetti and colleagues analyzed the role of the P2X7 receptor-dependent inflammasome in the progression of MASLD to MASH. The disease was induced by feeding wild-type and *P2rx7^−^/^−^* mice with a high-fat diet for 16 weeks. *P2rx7^−^/^−^* mice were less sensitive to developing MASH (1/7 vs. 4/7 of control) and displayed an attenuated expression of markers for oxidative stress, inflammation, and fibrosis. Furthermore, it was demonstrated that the deletion of P2X7 receptor expression prevented NLRP3 inflammasome activation by ATP in isolated liver sinusoidal endothelial cells (LSECs) from *P2rx7^−^/^−^* individuals [91]. A translational study by the same research group explored the role of the P2X7 receptor–NLRP3 inflammasome pathway in MASLD, MASH, and hepatitis C virus (HCV)-related hepatic disease using samples from 46 patients. According to the findings, P2X7 and P2X4 receptors, the NLRP3 inflammasome, and caspase-1 were highly expressed in liver biopsies from patients with HCV; pro-inflammatory interleukins, such as IL-2, IL-6, and TNF-**α**, were also elevated [91]. Although this study is interesting, it is inconclusive because it lacked a comparison against samples from healthy donors.

Baeza Raja and collaborators investigated which hepatic cellular types express the P2X7 receptor and the effects of its pharmacological inhibition in human cells and in a non-human primate model of liver injury [92]. In accordance with previous reports [91], this study demonstrated that the P2X7 receptor was overexpressed in human biopsies from MASH patients compared with healthy donors. Components related to NLRP3 and AIM2 inflammasomes were significantly increased in those biopsies. P2X7 receptor expression and inflammasome components were mainly localized in infiltrated immune cells and KCs in comparison to hepatocytes and HSCs [92], corroborating previous observations of the functional expression and pro-inflammatory actions of the P2X7 receptor in the KC line KUP5 [93].

Thus, the P2X7 receptor/NLRP3 pathway was characterized using in vitro isolated monocytes and KCs, with both cell types responding to the double stimulus of LPS + ATP with an evident increment in IL-1β release; this effect was abolished by co-administration of the P2X7 receptor antagonist SGM-1019. Interestingly, P2X7 receptor-mediated IL-1β release by KCs induced apoptosis in hepatocytes and activated HSCs in mice treated with LPS. In a preclinical trial using non-human primates, researchers assessed the importance of the P2X7 receptor as a therapeutic target. Monkeys were treated with CCl_4_ for four weeks with or without administration of the P2X7 receptor antagonist SGM-1019. The histological analysis of control livers revealed signs of hepatotoxic damage, characterized by hepatocyte ballooning, inflammation, and fibrosis; in contrast, these signs were counteracted by SGM-1019 [92]. These results strongly suggest that the P2X7 receptor is a suitable pharmacological target for therapeutics in a variety of hepatotoxic protocols.

Inflammatory responses mediated by the P2X7 receptor/NLRP3 pathway have also been described in other cell types besides KCs. Two-step NLRP3 activation was also tested in HSCs. Cultures from the HSC line LX-2 responded with a significant increase in IL-1β release when challenged with LPS + ATP; the response was abolished by preincubation with A438079, indicating that it was mediated by the P2X7 receptor. Furthermore, to investigate the dual cell-to-cell regulation between KCs and HSCs, LX-2 cell cultures were stimulated with conditioned medium (CM) from LPS-activated RAW 264.7 mouse macrophages. The CM induced an increment in transcripts for the inflammatory mediators *IL-1β*, *IL-18*, *P2rx7*, *Nlrp3*, and *Casp-1*; in the fibrotic markers *Acta2* and *Col1A1*; and, interestingly, in the release of ATP into the extracellular space, compared with CM from macrophages not activated with LPS. The release of IL-1β was also abolished by antagonizing the P2X7 receptor, evidencing the participation of the ATP/P2X7 receptor/NLRP3 pathway [94]; in the same study, the interplay between macrophages and HSCs orchestrated by the DAMP pair ATP/P2X7 receptor was also characterized. The sensitivity of HSCs to extracellular ATP was demonstrated in primary cultured mouse HSCs, where P2X7 receptor ligation induced NLRP3 inflammasome activity and supported inflammation in alcohol-mediated liver damage [95]. On the other hand, it has been described that exosomes from hepatocytes infected by the hepatitis B virus promoted HSC activation and, consequently, liver fibrosis; in the HSC line LX2A, it was shown that these responses were supported by the actions of METTL3 (N6-adenosine-methyltransferase 70 kDa subunit), which stabilized the *P2rx7* transcript by binding N6-methyladenosine (m6A). P2X7 receptor availability supported HSC activation [96].

In MASH, the P2X7 receptor has also been associated with the enhancement of metabolic oxidative stress (MOS), which is related to autophagy induction. In a study by Das and colleagues [97], MOS was induced by treatment with a methyl-choline-deficient diet and by administering the toxin bromodichloromethane (BDCM) in a diet-induced obesity (DIO) mouse background. DIO + BDCM induced MOS hallmarks in the liver, such as increases in 4-hydroxynonenal, tyrosine nitration, and 5,5-dimethyl-1-pyrroline N-oxide-nitrone adducts. Interestingly, increased transcript and protein expression of the P2X7 receptor were detected in both models. The effects of both treatments were attenuated in the knockout mouse for the cytochrome P-450 CYP2E1, one of the enzymes mediating MOS response. MOS-induced increments in the P2X7 receptor transcript were detected in hepatocytes, KCs, and LSECs [97]. MOS induction by both pro-oxidant diets also resulted in an upregulation of transcripts coding for autophagy-related markers (*Gabarap*, *Atg2a*, and *Lc3b*) and proteins of lysosomal translocation complex Hsc70 and Lamp2, which were also significantly attenuated in CYP2E1^KO^ mice. None of these changes were observed in mice lacking P2X7 receptor expression, suggesting that this receptor is an important player in the regulatory network of autophagy in MASH [97]. Furthermore, in addition to inflammation, the P2X7 receptor is an effector in a network of signals regulated by MOS through MASH in HSCs. Mechanistically, CYPE21 enhanced activity under oxidative stress, leading to high circulating leptin amounts that, in turn, upregulated P2X7 receptor expression. The receptor then triggered HSC activation and increased the intracellular concentration of glucose, thereby regulating the membrane localization of the Glut4 transporter and boosting the onset of fibrosis [98].

Recent studies characterizing the mechanisms that regulate P2X7 receptor expression in fibrotic liver have suggested that Kruppel-like factor 15 (KLF15), a transcription factor whose ablation potentiates steatosis and inflammation induced by high-fat diets [99], negatively modulates P2X7 receptor expression, and consequently, the P2X7 receptor/NLRP3 inflammatory axis [100]. On the other hand, a crosstalk between the TGF-β signaling and P2X7 receptor/NLRP3 pathways in the context of fibrosis has been described [101]. The authors assayed the feasibility of TGF-β receptor ALK5 inhibitors J-1155, J1156, and LY-2157299 to reverse thioacetamide-induced fibrotic damage. Indeed, the inhibitors prevented the increment in P2X7 receptor expression caused by thioacetamide, and hence, its inflammatory effects [101].

### 3.2. P2X4 Receptor

The P2X4 receptor is a member of the P2X subfamily, which is phylogenetically closer to the P2X7 receptor [86]. The P2X4 receptor is mainly homomeric and, compared with P2X7, it displays a significantly higher affinity for ATP (1–10 µM vs. 100–500 µm) and faster desensitization [102]. Experimental findings suggest that the P2X4 receptor plays an important role in the fibrogenic process.

The fibrogenic effect of BLD or the administration of a choline-deficient diet was assayed in P2X4 knockout mice (*P2rx4^−^/^−^*) and compared against wild-type individuals. Deletion or pharmacologic antagonism of P2X4 prevented collagen accumulation within the liver but did not affect steatosis, strongly suggesting that the P2X4 receptor is selectively expressed in liver MFs, the cells responsible for collagen I secretion. Interestingly, *P2rx4^−^/^−^* mice did not present any changes when fibrosis was induced by CCl_4_, suggesting that the role of the P2X4 receptor depends on fine-tuning mechanisms inherent to each experimental model. Moreover, upregulation of the *P2rx4* transcript was observed in biopsies from human patients with HCV, primary biliary cholangitis, primary sclerosing cholangitis, alcoholic cirrhosis, and NASH. Furthermore, in cultured human or mouse MFs, ATP stimulation of the P2X4 receptor upregulated the main activation marker, αSMA. Accordingly, siRNA downregulation of the P2X4 receptor reduced basal levels of this marker in MF cell lines and the fibrogenic phenotype by regulating cell adhesion, migration, and the balance between synthesis and degradation in the ECM. This study also demonstrated that the P2X4 receptor regulates lysosomal exocytosis phenomena in MFs, potentially leading to the secretion of molecules associated with the induction or maintenance of fibrosis [103]. Similar to the controversy with the P2Y6 receptor, the “contradictory” results regarding the role of the P2X4 receptor in the pathology of liver diseases could be context-dependent. Indeed, more experiments will be beneficial to elucidate this controversy.

In a model of alcoholic liver fibrosis (ALF) induced by ethanol plus CCl_4_ in mice, it was shown that the P2X4 receptor is upregulated in the liver; similar findings were observed in the HSC-T6 mouse cell line upon stimulation with acetaldehyde. In ALF-induced animals, administration of the P2X4 receptor antagonist 5-BDBD prevented fibrosis induction. In the HSC-T6 line, the main pathway activated by acetaldehyde was PI3K/AKT; silencing or pharmacological antagonism of the P2X4 receptor prevented HSC-T6 activation. Interestingly, activation of HSC-T6 cells induced by the conditioned medium of acetaldehyde-stimulated RAW267.4 macrophages was prevented by preincubating macrophages with the P2X4 receptor antagonist 5-BDBD [104]. Similar increments in P2X4 receptor expression in liver tissue were demonstrated by an independent study using the ALF model. This study also reported an enhancement in the expression of CD39 ectonucleotidase and total ATP content. Similarly, pharmacological treatments in whole liver and RAW267.4 macrophages showed that P2X4 receptor activity contributed to liver steatosis and the production of pro-inflammatory cytokines, with a concomitant regulation of CD39 expression. In the in vitro model, evidence showed that P2X4 receptor stimulation positively regulated the expression of NLRP3, caspase-1, and IL-1β inflammasome components; these changes were correlated with increased intracellular Ca^2+^ responses. Furthermore, the conditioned medium of RAW267.4 macrophages stimulated with ethanol increased apoptosis in the hepatocyte line AML-12 [105].

On the other hand, HCV infection of HEK293 cells overexpressing the P2X4 receptor regulated the expression of the antioxidant enzymes HO-1 and Cu/Zn SOD, boosting the expression of TNF-α, TGF-β, angiotensin II, and components of the ECM, namely laminin and elastin [106]. Since the participation of other P2X receptors in liver fibrosis is unknown, the role of the P2Y receptor in fibrosis will be discussed below.

### 3.3. P2Y2 Receptor in Liver Fibrosis

The P2Y2 receptor belongs to the P2Y1-like subfamily of P2Y receptors. It is coupled to Gq proteins and thus associated with the turnover of phosphoinositides and Ca^2+^. The order of potency of natural and synthetic ligands for the human P2Y2 receptor is as follows: agonists MRS2698 = UTP ≥ ATP > Ap4A; antagonists AR-C118925 > PSB-11415 [107]. UTP and ATP naturally occur in the cell and may function as DAMPs or as autocrine/paracrine mediators [108,109]. The P2Y2 receptor plays a pivotal role in hepatic cell renewal and cell proliferation [110,111,112]. In a physiological state, this receptor is located mainly in the perivascular regions of the central and portal veins. In the fibrotic liver induced by CCl_4_, the P2Y2 receptor loses its zonal distribution, spreading throughout the entire hepatic parenchyma and exhibiting increased expression. This increase is related to an exacerbation of P2Y2 receptor responses in fibrotic hepatocytes [113].

The P2Y2 receptor is the only receptor involved in the induction of acute hepatitis with concanavalin A in a murine model. Ayata et al. demonstrated that its expression in bone marrow is crucial for neutrophil infiltration in the damaged liver. They also showed that activation of this receptor potentiates the TNF-**α** cell death response in hepatocytes. This effect was counteracted by preincubation with suramin, a classical antagonist of the P2Y2 receptor, which also reduced hepatic necrosis and circulating transaminase levels [114].

In the context of metabolic syndrome, Merz and colleagues found few signs of metabolic syndrome in adipose tissue from *P2ry2^−^/^−^* mice consuming a high-fat diet. The mice gained less weight, and their liver and adipose tissue occupied less volume than in wild-type mice. Plasma cholesterol and C-peptide levels were lower, and insulin resistance was improved in KO mice. Findings demonstrated that interfering with P2Y2 signaling prevents accumulation of immune cells in this model, reducing inflammation of adipose tissue and improving the metabolic phenotype [115].

In ALD, a blockade of the P2Y2 receptor with suramin prevented liver damage and lipid accumulation and decreased CD39 levels in mice. Protection with suramin proved to be dose-dependent. The best performance was identified at a high dose (20 mg/kg). This dose effectively damped circulating transaminases, free fatty acids, and inflammatory cytokines [116]. In isolated mouse hepatocytes and the RAW264.7 macrophage cell line, inhibition of the P2Y2 receptor with suramin or siRNA decreased the expression of IL-6, IL-1β, TNF-α, and CD39. In addition, exposure to 100 µM of ethanol increased P2Y2 receptor, CD39, and ATP concentrations [116]. Liu et al. suggested that the increase in CD39 ectonucleotidase represented a negative feedback mechanism for ATP-induced changes, as hydrolysis of the nucleotide limits P2Y2 receptor activation. In 2022, Liu more deeply explored the mechanism of liver inflammation mediated by the P2Y2 receptor in ALD. The authors found that the P2Y2 receptor regulated inflammation through the EGFR-ERK1/2 pathway and demonstrated that blockade with suramin or P2Y2 receptor silencing with siRNA attenuated ethanol-induced hepatocyte apoptosis and decreased pro-inflammatory cytokines, such as TNF-α and IL-1β [117].

In a murine model of hepatic fibrosis induced by CCl_4_, our group evaluated the role of the P2Y2 receptor. Similar to the findings above regarding the loss of zonal distributions of P2Y2 receptors in livers from CCl_4_-treated mice, we found that fibrotic hepatocytes presented exacerbated ERK phosphorylation in response to UTP. Furthermore, using microarrays, we observed that P2Y2 receptor activation induced significant changes in gene expression in fibrotic hepatocytes compared to healthy hepatocytes, including transcripts coding for DNA repair proteins [113]. Hepatocytes isolated from fibrotic mice showed that P2Y2 receptor activity prevented the formation of histone γH2AX foci induced by etoposide, a drug that promotes double-strand DNA damage; this effect correlated with the upregulation of a cluster of genes associated with double-strand DNA repair. We also found that the response mediated by the P2Y2 receptor required a crosstalk with hypoxia-induced factor 1 (HIF-1α) signaling [118]. The P2Y2 receptor is a multifaceted mediator in hepatic fibrosis and other inflammatory liver diseases. Overexpression and activation of this receptor in hepatic parenchyma not only facilitate inflammatory and proliferative responses, but also play a crucial role in the preservation of genomic integrity in hepatocytes by activating DNA repair mechanisms mediated by HIF-1α [118]. Pharmacological regulation of P2Y2 receptor signaling is a promising therapy for modulating the progression of fibrosis and other chronic hepatic diseases.

In another model of metabolic syndrome, Cano-Martínez et al. showed that treatment with resveratrol and quercetin downregulated the expression of the P2Y2 receptor, TLR4, and neutrophil elastase. These combined treatments reduced collagen deposition, hepatic fibrosis, and liver apoptosis. Improvement was detected especially with the normalization of circulating transaminases [119]. In this context, Dusabinama et al. studied the effect of a high-fat diet on *P2ry2^−^/^−^* mice; they found that P2Y2 receptor deficiency improved insulin resistance and favored the decline in serum levels of insulin and transaminases, as well as decreased expression of fatty acid synthesis mediators, such as fatty acid synthase and stearoyl-CoA desaturase 1. In addition, P2Y2 receptor deficiency enhanced the activity of AMP-activated protein kinase (AMPK), improving fatty acid oxidation [120].

In the context of inflammation caused by bacterial agents, Arunachalam A. et al. evaluated the impact of a sepsis model in *P2ry2^−^/^−^* mice. They reported that KO mice were protected against mortality and systemic cytokine storms. P2Y2 receptor activation depleted serum arginine, a vital part of the immunological defense against bacteria. In addition, *P2ry2^−^/^−^* mice presented more efficient bacterial elimination. Furthermore, *P2ry2^−^/^−^* mice showed attenuated inflammation and liver injury, as well as bacteremia, enhancing hepatocyte survival. Another discovery was that *P2ry2^−^/^−^* mice exhibited reduced hepatic MMP-9 expression [121]. It has been reported that high levels of MMP-9 promote liver fibrosis, inflammation, and macrophage recruitment, leading to epithelial–mesenchymal transition [122].

In the next two sections, we will discuss the remaining P2Y1-like receptors and the other subfamily of P2Y12-like receptors.

### 3.4. P2Y1-like Receptors (P2Y1, P2Y2, P2Y4, P2Y6, P2Y11)

In 2004, Dranoff et al. found that P2Y receptors, specifically UDP-sensitive P2Y6, increased procollagen-1 transcript levels by three times in rat MFs. The authors also found that P2Y2 and P2Y4 receptors were expressed in qHSCs [123]. Several years later, Yuan et al. reported in an ASH murine model that inhibition or silencing of P2Y6 receptor expression reduced liver inflammation and steatosis by decreasing TNF-α, IL-1β, and IL-6 after interrupting the p38 MAPK pathway activated by UPD-mediated calcium release [124]. The response induced by UDP was similar in RAW 264.7 cells. These results suggested that the P2Y6 receptor could act as a possible therapeutic target, but these results were not reproduced by Nishiyama’s group. Instead, they found that in *P2ry6^−^/^−^* mice, hepatic steatosis was not alleviated, but rather aggravated, with an increase in serum AST and C-C motif chemokine 2 mRNAs in choline-deficient rats treated with a high-fat diet. These findings suggested that the P2Y6 receptor did not contribute to disease progression [125]. Unlike the study conducted by Yuan, Nishiyama used a NASH model; this difference might account for the discrepancy in results. Certainly, more experiments are needed to define the role of the P2Y6 receptor in hepatic maladies since it is very probable that the importance of this receptor could be dependent on pathological context.

On the other hand, in normal human cholangiocytes and cholangiocarcinoma cell lines (HuCCT1, Mz-Cha1, and F258), it was shown that stimulation of the bile acid receptor TGR5 induced Gs- and Gq-mediated responses. The increase in cAMP activated PKA, which phosphorylated and activated cystic fibrosis transmembrane conductance regulator (CFTR) Cl^−^ channels, whereas intracellular Ca^2+^ mobilization promoted ATP/UTP secretion in an autocrine manner, transactivating P2Y2 and P2Y4 receptors. In turn, both purinergic receptors enhanced intracellular Ca^2+^ dynamics, thereby activating TMEM16 and, consequently, Cl^−^ mobilization. Cl^−^ efflux by CFTR or TMEM16 was exchanged for HCO_3_^−^ through AE2 transporters in cholangiocytes [126]. This pathway may regulate cholangiocytes during fibrosis progression.

Recently, our group identified the expression of the P2Y1 receptor in qHSCs isolated from mice; P2Y1 receptor activation by ADP induced a fast Ca^2+^ transient that was completely dependent on release from intracellular stores. Long-term stimulation with 10 µM ADP delayed HSC transdifferentiation to MFs and prevented the nuclear translocation of YAP [127]. We hypothesize that the P2Y1 receptor could play a protective role that maintains the quiescent status and prevents the onset of fibrosis.

### 3.5. P2Y12-like Receptor (P2Y12, P2Y13, P2Y14)

Finally, we will mention the role of P2Y12-like receptors in liver fibrosis. The first report relating hepatic inflammation to the P2Y12 receptor was in 2010. Sullivan and collaborators [128] tested the hypothesis that platelet-dependent coagulation contributes to cholestatic damage of the liver. In a mouse model of alpha-naphthyl isothiocyanate-induced cholestasis, conglomerates of platelets were found around necrotic foci within the liver. Systemic administration of the P2Y12 receptor antagonist clopidogrel prevented an increase in serum alanine aminotransferase, and reduced hepatocyte necrosis and neutrophil infiltration in the liver. These results were interpreted as a probe that platelets contribute to inflammatory damage in acute cholestasis [128].

In the context of type II diabetes mellitus, L. Li et al. highlighted the importance of the P2Y12 receptor in liver inflammation. In rats with this condition, the P2Y12 receptor is overexpressed, and levels of NLRP3, active caspase-1, and interleukin-1β are elevated. However, when shRNA targeting P2Y12 was used, the expression of NLRP3, caspase-1, and IL-1β decreased. Furthermore, serum levels of cholesterol and triglyceride were reduced. Liu’s group concluded that P2Y12 may contribute to hepatic inflammation by activating the NLRP3 inflammasome, and also may be involved in the regulation of hepatic glucose and lipid metabolism, indicating that the P2Y12 receptor is a possible target for the treatment of this disease [129].

The P2Y13 receptor in the KUP5 macrophage cell line was involved in pro-inflammatory action mediated by IL-6 upon LPS treatment. In this context, concomitant treatment with the P2Y13 receptor antagonist MRS2211, as well as genetic knockdown of this receptor, significantly inhibited LPS-induced IL-6 production but without affecting TNF-α production. Furthermore, it was demonstrated that the effects of LPS on macrophages involved ATP release, revealing an autocrine–paracrine feedback loop that contributed to KC activation [130].

Duparc et al. subjected wild-type and P2Y13^−^/^−^ mice to an obesogenic diet containing high levels of fat, sucrose, and cholesterol. Results showed that KO mice display higher levels of IL-6 and monocyte chemoattractant protein (MCP-1) in adipose tissue; similarly, circulation levels of IL-6, MCP-1, TNF-α, and transaminases were also enhanced, suggesting liver damage and systemic inflammation. Furthermore, increased expression of profibrotic genes, such as alpha-SMA and Tgfb1, was shown in P2Y13^−^/^−^ mice [131]. In summary, deficiency of the P2Y13 receptor promoted systemic inflammation and aggravated steatosis and hepatic fibrosis, suggesting a regulatory role in the adipose tissue–liver axis.

In another report, Mederacke et al. [132] explored DAMP/DAMP-receptor pairs produced by damaged hepatocytes and targeted HSCs to induce their activation in a murine model. The authors demonstrated that UDP-glucose acts as a DAMP and a ligand for the P2Y14 receptor in damaged hepatocytes. Activation of the P2Y14 receptor promotes the ERK signaling pathway and mediates HSC transdifferentiation to MFs. KO mice for this receptor showed significantly reduced fibrosis in different models of liver injury [132]. Recently, a new family of antagonists against the P2Y14 receptor was designed using computational approaches; the most potent (IC50 = 0.026 nM) and metabolically stable molecule was denominated as HDB-1 (component 47). HDB-1 was evaluated in in vivo models of liver fibrosis, including toxicity induced by CCl_4_ and a choline-deficient high-fat diet, as well as in LX2 cells and primary cultured HSCs. The pharmacological inhibition of the P2Y14 receptor with HDB-1 significantly reduced liver fibrosis by suppressing HSC activation through the PKA/Raf1/MEK/ERK pathway [133].

The evidence described show that P2Y receptors play a pivotal, complex role and are dependent on the context of hepatic physiology and pathology. Meanwhile, receptors P2Y6 and P2Y14 are associated with pro-inflammatory and profibrotic processes; some contradictory results between different experimental models highlight the importance of the disease context and methodological differences. On the other hand, P2Y1, P2Y2, and P2Y14 receptors could perform protective or modulating functions, especially regarding the maintenance of qHSCs and modulating biliary activity.

**Figure 2:**
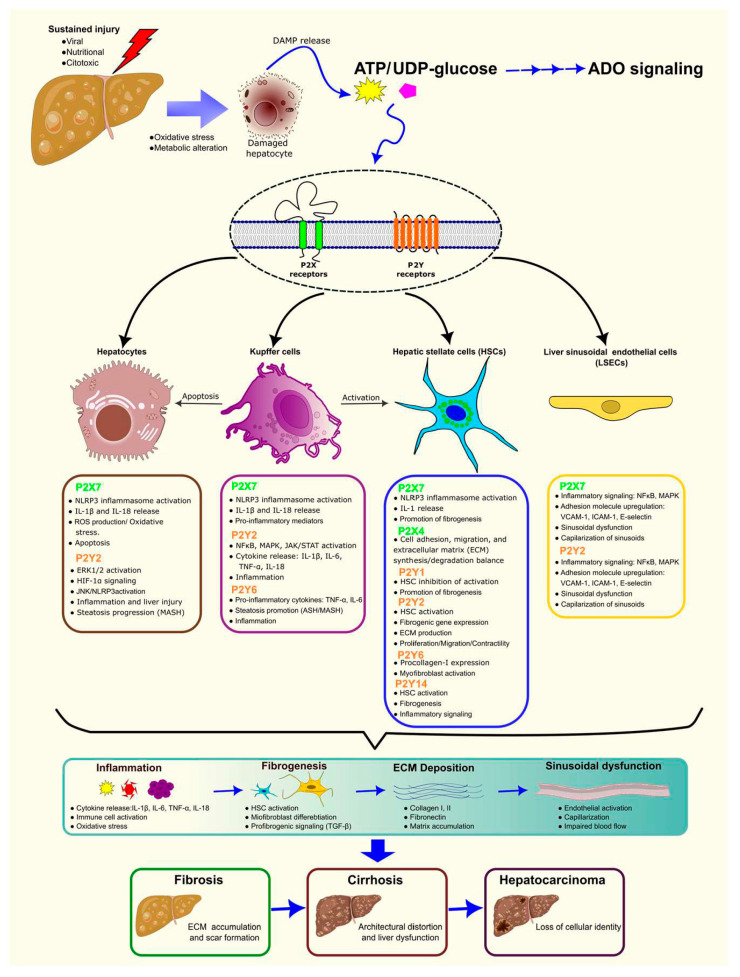
Cell-specific purinergic signaling networks during chronic liver disease progression. Extracellular ATP and UDP-glucose released from injured hepatocytes activate P2X and P2Y receptors expressed by HSCs, KCs, hepatocytes, and LSECs. Receptor activation triggers distinct intracellular signaling pathways that regulate inflammatory responses, HSC transdifferentiation, extracellular matrix production, tissue remodeling, and metabolic homeostasis. ATP hydrolysis by ectonucleotidases modulates these responses through the sequential generation of ADP, AMP, and adenosine. Depending on the receptor subtype and cellular context, purinergic signaling may exert either profibrotic or protective effects, ultimately influencing the progression from chronic liver injury to fibrosis, cirrhosis, and hepatocarcinoma. Images of livers and cells were sourced and adapted from NIH BioArt and Biocoins (https://bioart.niaid.nih.gov/ and https://bioicons.com/).

Collectively, these findings demonstrate that purinergic signaling is a highly coordinated and context-dependent regulatory network in the liver. The biological outcome of receptor activation depends not only on the receptor subtype but also on the responding cell type, the extracellular nucleotide involved, and the stage of liver disease. Figure 2 summarizes the principal P2-receptor-mediated signaling pathways and their contribution to inflammation, fibrosis, cirrhosis, and hepatocellular carcinoma, providing an integrated overview of the mechanisms discussed throughout this review.

## 4. Context Determines Purinergic Outcomes in Liver Fibrosis

Nonetheless, hepatic fibrosis is a well-defined pathological state: its generation, installation, persistence, or potential reversibility is dependent upon the particular circumstances surrounding the experimental model (see Table 1), initial damage factors, ontogenic state, and associated physiological conditions (sex, species, microbiome) within a given biological system. Indeed, this context dependency will have a relevant impact on the role played by the purinergic system during the fibrotic process. Considering each of the liver cell types, the potential of ATP and UDP-glucose as DAMPs to influence the progression of fibrosis is contingent upon the changes and evolution of the corresponding purinome: the equilibrium between ATP and ADO signaling, the corresponding expressed receptors, the ligand proportion according to ectonucleotidase activities, and the intracellular signaling pathways activated in each cellular population. These considerations should be considered for any experimental model as well as for any potential translational application.

Given the great complexity in the interplay of purinergic signaling elements in the multiple responses of cellular liver types involved in the fibrotic process, the appearance of apparent contradictions or inconsistencies in the role played by ATP receptors is not unexpected. For example, Le Guilcher et al., 2018 [103], reported a different relevance of the P2X4 receptor during onset and fibrotic development according to the experimental system studied: (a) genetic-deficient animals, (b) hepatotoxic treatments (CCl_4_, LPS), (c) surgical procedures (BDL), (d) experimental diets (methionine/choline-deficient diet), and (e) cell lines. In this context, the fibrotic role played by the P2Y6 receptor was supported in MFs and KCs [123,124], whereas in mice lacking the expression of this receptor, it was reported to fail in improving liver injury and inflammation in non-alcoholic steatohepatitis [125]. Indeed, a putative explanation is that the outcome of each report is dependent on the cellular context in each experimental design.

Available literature regarding purinergic receptors and liver fibrosis was explored in previous sections; in the next paragraphs, we discuss the purinergic action alongside the fibrogenic process. As aforementioned, liver fibrosis is reversible and represents a strategic window for potential therapeutic interventions.

In the different types of liver damage, the first injured cells are hepatocytes [90]. A common event in liver injury is MOS that, among other effects, induces enhanced pro-oxidant reactions, including lipid peroxidation, with consequent membrane disruption and cell death [134]. In hepatocytes and most eukaryotic cells, the cytosolic concentration of ATP is high (between 5 and 10 mM) [135,136]; when the cytosolic content is released, the concentration of ATP is enough to activate any purinergic receptor present in the neighboring cells, including the low-affinity P2X7 receptor (Kd 500 uM to 1 mM) [82]. Thus, this purinergic signaling triggers diverse and complex responses.

Evidence supports that extracellular ATP acting through purinergic receptors is one of the main factors participating in different stages of the fibrotic process, including MOS and autophagy. For example, in the MASH model, high levels of leptin promote P2X7 receptor overexpression in hepatocytes, KCs, and LSECs [97]. This receptor functions as an effector of MOS signaling that, in response to high levels of extracellular ATP, induces the expression of autophagy-related genes. Autophagy protects hepatocytes but boosts HSC activation by a mechanism that has not yet been clarified [97] (see [137] for a comprehensive review about autophagy and liver disease); these events indicate a relevant role of the P2X7 receptor in the onset of hepatic disease. In addition, the establishment of an inflammatory environment during fibrosis is recognized as relevant. It has been reported that chronic inflammation is an important contributor to fibrosis onset, as well as to the progression of cirrhosis [138]. Abundant evidence supports the role of the P2X7 receptor in the induction and maintenance of inflammation in different experimental models, such as alcohol consumption [90], MASLD and MASH [91], and CCl_4_ administration [92]. The cell types orchestrating an ATP-induced inflammatory response in this stage of fibrogenesis are LSECs [91], KCs [92,93] and HSCs [94,95]. The main event supporting the inflammatory response is the assembly of the NLRP3 inflammasome promoted by P2X7-receptor-mediated Cl^−^ efflux [90,91,92,93,94]. Importantly, it is well established that NLRP3 inflammasome activation induces hepatocyte pyroptosis, inflammation, and fibrogenesis, and boosts HSC activation [139] (Figure 1). NLRP3 assembly, associated with ATP release, could establish a positive inflammatory response and a fibrotic feedback loop, considering that the P2X7 receptor does not suffer desensitization in the presence of its agonist [87]. In addition, the P2X4 receptor through NLRP3 activation [105], the P2Y2 receptor activating the EGFR-ERK pathway [117] and the P2Y13 receptor [130] have also been related with inflammation in a fibrosis context.

A variety of studies support the notion that HSCs are the main source of fibrogenic MFB cells [140,141]; indeed, transdifferentiation of HSCs is of primary importance in the onset and maintenance of liver fibrosis. Although it is well recognized that HSC activation is importantly driven by the cooperative action of messengers such as TGF-β and PDGF [142], the contribution of DAMP/DAMPR in this process has recently been recognized, particularly UDP-glucose released by injured hepatocytes acting through the P2Y14 receptor in quiescent HSCs through a pathway that involves ERK signaling [132]. Regarding the relevance of roles played by MFBs during liver fibrosis, it has been reported that P2X4 receptor activity supports the fibrotic phenotype in MFBs. Its actions include upregulation of αSMA expression along with an enhanced synthesis of the ECM [103]. Potentially, the specific expression and action of the P2X4 receptor in MFBs may be exploited in clinical trials.

From this perspective, the relevance is clear of the cell specificity and temporal precision of ATP/P2 receptors in the whole fibrotic process. Another dimension of complexity is added by the concomitant conversion of ATP in its dephosphorylated metabolites, mainly in ADO by a set of ectonucleotidases. Importantly, it was reported that ALF induced overexpression of CD39, an enzyme with the ability to synthesize ADP and AMP from ATP [105]. Moreover, reported evidence has shown that the P2Y2 receptor supports CD39 expression, and that ethanol can upregulate the expression of this ectonucleotidase; hence, it was suggested that this increase limits the availability of extracellular ligands for the P2Y2 receptor [116]. On the other hand, MFBs but not quiescent HSCs express ENTPD2 [143]. Thus, it is clear that release of extracellular ATP can favor ADP synthesis and, eventually, ADO production. In an interesting purinergic balance, ADO via A2A receptors can function as a profibrotic factor [144]. Certainly, adenosinergic signaling in cellular components of the liver should be reviewed in detail in future analysis.

Collectively, these findings underscore the complexity of purinergic signaling during chronic liver disease. Although several P2X and P2Y receptors represent promising therapeutic targets, their physiological functions and context-dependent effects suggest that selective, receptor-specific interventions will be required to maximize therapeutic benefit while minimizing undesirable effects (Table 2).

## 5. Concluding Remarks

Due to the complexity of the fibrotic process, it is necessary to establish the cellular context in which it occurs. However, regardless of the affected organ or tissue, fibrosis is a problem of cellular adaptation to its surroundings since the extracellular milieu determines correct cell function. Fibrosis is a distinguishing feature in the progression of hepatic disease that may culminate in cirrhosis or cellular hepatocarcinoma. Indeed, a vast number of intermediaries and molecular messengers from diverse cell types interact to initiate, regulate, or revert fibrotic development. In this context, the role of ATP not only as an energetic metabolite but also as a cellular ligand is recognized as a key factor in adaptations to stressful circumstances associated with cellular damage. Hence, fibrosis is influenced not only by ATP-activated G-protein-coupled receptors and ligand-activated ion channels, but also by other purinergic signaling elements associated with ATP multistep enzymatic transformation. Therefore, it can be considered that purinergic signaling is a stage-dependent and cell-specific communication network that can amplify liver damage and determine fibrosis resolution versus persistence, which may become therapeutically actionable only if receptor targeting is matched to disease etiology, fibrosis stage, and cellular context. Future pharmacological and therapeutic approaches will focus on gaining a better understanding of purine’s strategic role in the fibrotic process within the liver and other organs.

## Figures and Tables

**Table 1:** Animal models for the study of fibrosis.

Model	Method	Species	Duration (Weeks)	Degree of Induced Fibrosis	References
Chemical					
Ethanol	Mixed with water or liquid diets Intragastric feeding	Rat/Mouse	4–12 4–16	Difficult to induce liver fibrosis Need for a second hit to induce fibrosis	[17] [41] [42]
CCl_4_	Intraperitoneal injection Inhalation	Rat/Mouse	4–12	**	[43]
Thioacetamide (TAA)	Intraperitoneal injection Subcutaneous injection Subcutaneous injection Oral administration	Rat Macaque Marmoset	12–13 11 2–16	**	[44]
Dimethylnitrosamine (DMN) N-nitrosodiethylamine (DEN)	Intraperitoneal injection Intraperitoneal injection	Male SD rats Rat/Mouse	4–8 4–6	*** ***	[45] [46]
Diet					
Methionine- and choline-deficient (MCD) diets High-fat diet (HFD) Western diet (WD) Fast food diet (FFD) Choline-deficient l-amino acid-defined (CDAA) Choline-deficient l-amino acid-defined high-fat diet (CDAHFD)	Feeding Feeding Feeding Feeding Feeding Feeding	Mouse Mouse Ossabaw pig Mouse Rat/Mouse Rat/Mouse	5–8 24–25 16 30 12 6–9	* * * ** * **	[47] [48] [38] [49] [50] [51]
Surgical					
Bile duct ligation (BDL)	Double ligation of the common bile duct	Rat/Mouse	3–4	**	[52]
Immunity					
Schistosoma (*Schistosoma japonicum*) Virus (Human hepatitis B) Porcine serum Concanavalin A	Subcutaneous injection Tail vein injection Intraperitoneal injection Intraperitoneal injection	Mouse Mouse Rat Mouse	8 4–5 16–24 4–8	** ** * *	[53] [54] [55] [56]
Transgene					
Gnmt^−^/^−^	Genetically modified mice Genetically modified mice Genetically modified mice Genetically modified mice	Mouse Mouse Mouse Mouse	12 8–14 12 20	** ** ** **	[57] [58] [59] [60]

Degree of induced fibrosis: (*) mild, (**) moderate, and (***) severe.

**Table 2:** Therapeutic targeting of purinergic receptors in liver fibrosis: experimental evidence, pharmacological strategies, expected benefits, and translational considerations.

Receptor	Ligand	Cell Type	Experimental Model	Evidence Level	Available Inhibitor/Antagonist	Expected Therapeutic Benefit	Major Translational Considerations	Key Findings	Refs.
P2X4	ATP	HSCs and MFBs	BDL, choline-deficient diet and ALF	KO and antagonism In vivo and in vitro	5-BDBD	Remission of MF phenotype and prevention of HSC activation	Unknown	P2X4 maintains MF characteristics	[104,105]
P2X7	ATP	KCs and infiltrated macrophages	CCl_4_ in mice and non-human primates; MASLD and MASH in mice	In vitro, in vivo, and preclinical trial in monkeys	A4380789 SGM-1019 P2X7^−/−^	Attenuate inflammation and prevent fibrosis-associated cellular changes	Experimental evidence supports a tumor-suppressor role for the P2X7 receptor	P2X7 inhibition or silencing dampened inflammation	[89,90,91,92]
P2Y1	ADP	HSCs	Mechanotransduction-induced HSC activation	In vitro	MRS2500	Suppression of HSC activation and fibrogenesis	Unknown	P2Y1 antagonism attenuates HSC activation	[127]
P2Y2	ATP/ UTP	Hepatocytes	Acute hepatitis induced by acetaminophen	In vivo	P2Y2^−/−^ mice, Suramin	Reduction in inflammation and TNF-a-dependent cell death	Potential impairment of regenerative responses	P2Y2 receptor inhibits cell survival genes and promotes TNF-a-dependent cell death	[114]
		Whole-liver tissue and isolated hepatocytes in mice	ALD	Preclinical (in vivo/in vitro)	Suramin P2Y2 receptor knockdown	Reduction in steatosis and inflammation	Potential impairment of innate immune defense	P2Y2 receptor regulates production of pro-inflammatory cytokines in the onset of liver fibrosis	[116,117]
		Isolated fibrotic mice hepatocytes	CCl_4_ -treated mice	In vitro	UTP	Reduction in DNA damage, potentially avoiding genomic instability and carcinogenesis	Unknown	P2Y2 signaling dampened DNA damage induced by etoposide in fibrotic hepatocytes	[113]
P2Y6	UDP	HSCs	Rat	In vitro	MRS2578	Reduction in HSC activation and collagen production	Interference with physiological tissue remodeling	P2Y6 receptor regulates procollagen-1 expression in activated HSCs (MFs)	[123]
		Whole liver	ALD	Preclinical (in vivo/in vitro)	MRS2578 P2Y6 knockdown	Reduction in inflammation and steatosis	Independent studies found controversial results [125]	P2Y6 receptor boosts pro-inflammatory responses in ALD	[124]
P2Y12	ADP	Platelets and whole liver	ANIT-induced cholestasis mice	Preclinical (in vivo)	Clopidogrel	Attenuation of acute liver injury	Bleeding risk due to platelet inhibition	Platelet P2Y12 antagonism mitigates acute liver injury	[128]
P2Y13	ADP	KUP5 macrophage cell line	LPS-treated mice	Preclinical (in vivo/in vitro)	MRS2211 and siRNA	Suppression of pro-inflammatory responses	Possible disruption of immune homeostasis	P2Y13 triggered IL-6 production in liver-infiltrated macrophages	[130]
		Adipocytes and liver tissue	MASH mouse	Preclinical (in vivo)	*P2y13*^−^/^−^ mice	Improvement of steatosis and insulin resistance	Potential metabolic consequences	P2Y13 regulated metabolic changes related with fibrogenesis	[131]
P2Y14	UDP-glucose	Hepatocytes and HSCs	CCl_4_, BDL, and APAP-induced acute liver injury in mice	In vivo, mice	PPTN HDB-1	Attenuation of fibrosis and inflammation	Unknown effects on tissue repair	UDP-glucose released by damaged hepatocytes mediates HSC activation through the P2Y14 receptor	[132]
		HSCs	CCl_4_ and MASH mice	Preclinical (in vivo)	PPTN HDB-1	Reduction in fibrosis	Unknown long-term safety profile	P2Y14 antagonism reduces HSC activation and fibrosis	[133]

## Data Availability

No new data were created or analyzed in this study.
